# A theoretical review on the role of positive emotional classroom rapport in preventing EFL students’ shame: A control-value theory perspective

**DOI:** 10.3389/fpsyg.2022.977240

**Published:** 2022-12-01

**Authors:** Chunxiao Kang, Jianxiao Wu

**Affiliations:** ^1^School of International Education, Zhejiang Technical Institute of Economics, Hangzhou, China; ^2^School of Humanities and Management, Zhejiang Chinese Medical University, Hangzhou, China

**Keywords:** positive emotional classroom rapport, shame, EFL student, EFL teacher, control-value theory

## Abstract

**Background:**

Second/foreign language teaching has been considered as a dialogic and interactive job in which teachers’ and students’ emotions and behaviors are closely connected to each other. When there is a harmonious and positive relationship between the teacher and students in the classroom, many favorable academic outcomes may emerge. A bulk of research has endorsed the power of positive emotional classroom rapport in EFL contexts. However, its role in preventing negative students’ emotions like shame, as an achievement emotion, in terms of perceived control and value tasks has rarely (if any) caught scholarly attention.

**Objective:**

This study aimed to provide insights into the role of emotions in L2 education and the way students’ shame can be prevented or curbed in light of a positive emotional classroom rapport.

**Method/Design:**

This article systematically reviewed the theoretical and empirical underpinnings of EFL teachers’ positive emotional classroom rapport and students’ shame in light of the control-value theory.

**Results:**

In this research, it was asserted that by building a positive emotional classroom rapport EFL teachers can block and even eliminate students’ shame.

**Implications:**

The study offers practical implications to EFL teachers, trainers, principals, and researchers by increasing their knowledge and abilities in managing psycho-emotional mechanisms and factors and enriching interpersonal aspects of EFL education.

## Introduction

Language teaching and learning are two complicated processes with interactive and interpersonal natures (Dobransky and Frymier, 2004). Hence, it is unwise to commit oneself to the belief that teachers and students’ classroom behaviors, performances, and practices are totally separated from each other (Nathan, 2018; Zhu and Urhahne, 2021). Second/foreign language classroom is a representation of a society in which teachers and students are constantly in mutual interactions and talks to solve academic challenges (Zhou, 2003; Sun et al., 2022). In such an interactive milieu, both the teacher and students affect each other emotionally in positive or negative manners (Luo et al., 2020; Shao and Parkinson, 2021). If the relationship is negative, unfavorable outcomes may emerge in academia such as stress, tension, anxiety, demotivation, and the like (Hashemi, 2011; Alnuzaili and Uddin, 2020). In contrast, when the teacher and students form a positive relationship in the classroom, a harmonious atmosphere will be created that may lead to many positive academic outcomes like motivation, interest, passion, enjoyment, and so forth (Ibarra, 2014; Zhu and Urhahne, 2021). This optimal relationship is technically known as “classroom rapport,” which is the top priority of many educational practitioners all around the globe (Frisby and Martin, 2010). The term refers to a positive and constructive learning atmosphere that facilitates both teaching and learning processes and entails joy, mutual trust, respect, and connection (Reyes and Von Anthony, 2020). In order to establish a positive emotional classroom rapport in L2 classes, EFL teachers must take into account their students’ interests, needs, values, beliefs, ideas, and desires to form a friendly and democratic academic setting (Wilson et al., 2010).

A growing body of research shows that building a positive emotional classroom rapport brings about self-disclosure (Mazer et al., 2007; Hosek and Thompson, 2009), immediacy (Frymier and Houser, 2000; Rocca, 2008), clarity (Chesebro and McCroskey, 1998), motivation (Koca, 2016; Zheng et al., 2021), academic success (Estepp and Roberts, 2013; Jimerson and Haddock, 2015), confirmation (Goodboy and Myers, 2008), sense of fulfillment (Nathan, 2018), instructional communication competence (Worley et al., 2007), and academic engagement (Zhu and Urhahne, 2021). These studies signify that EFL teachers’ interpersonal communication behaviors play a crucial role in students’ learning processes. In addition to these positive academic outcomes and constructs, teachers’ ability to establish a positive emotional classroom rapport can go a step further and prevent negative stressors and hindrances of EFL education. One of such negative constructs among EFL students that can be curbed or removed through classroom rapport is shame, which is itself an overlooked construct in L2 research due to its elusive nature, inherent complexity, difficulty to define, and overlaps with other terminologies (Galmiche, 2018). The existing studies on student’s shame have only focused on its causes and consequences (Teimouri, 2017) and offered practical agendas and strategies for EFL teachers and students to stop this debilitative emotion and replace it with positive ones remained under-researched. The mechanisms, such as classroom rapport, *via* which shame as an achievement emotion can be replaced with positive emotions still needs to be argued from a control-value perspective. In light of CVT, control and value appraisals of learning activities are two main determining factors of emotions like shame (Pekrun and Perry, 2014).To fill this gap, the present study aimed to provide a theoretical analysis of the role of EFL teachers’ ability to build a positive emotional classroom rapport in preventing or removing students’ shame in the classroom *via* a CVT perspective.

## Background

### The interpersonal aspect of teaching

Language teaching and learning is known as a multidimensional and interpersonal field, whose purpose is to motivate students to get engaged in the process of learning rather than simply expose them to a heavy load of information (Barr, 2016; Xie and Derakhshan, 2021). The overall classroom climate and teacher-students’ interactions have been proven influential in students’ learning and development (Norton, 2008). A significant manifestation of interpersonal and prosocial bonds in EFL education is classroom rapport which involves mutual trust, understanding, and respect between the teacher and students in the classroom (Barr, 2016). Rapport building is one of the core features of effective teachers (Catt et al., 2007; Worley et al., 2007). Nowadays, teaching is regarded as a rapport-intensive profession owing to teachers’ and students’ relational goals and their constant attempts to enhance active participation in the process of teaching and learning (Frymier, 2007). There are different ways to enrich the interpersonal aspect of EFL education including (1) forming a positive emotional classroom climate, (2) using confirming behaviors, (3) wise responding to students’ questions, (4) praising students’ performance, (5) adjusting teaching style, (6) using humor in the class, and (7) using self-disclosure (Ellis, 2004; Brookfeld, 2006; Frymier et al., 2007; Hosek and Thompson, 2009; Barr, 2016; Xie and Derakhshan, 2021). The use of each of these strategies communicates teachers’ sense of care, concern, and interest to students, which in turn, raises their functionality in academic domains.

### The conceptualizations of positive classroom rapport

The concept of classroom rapport is a positive interpersonal factor, which pertains to the affective link between teachers and their students that is formed on the basis of mutual understanding, care, and respect (Lammers and Byrd, 2019). It is a close bond between the teacher and students that allows them to work cooperatively in the classroom (Culpeper and Kan, 2020). The concept of rapport has been conceptualized as a general impression of the teacher incorporating a reciprocal and prosocial connection in the class that leads to an enjoyable interaction (Ryan et al., 2011). Therefore, it is a perceived feature that reflects teachers’ communication in the eyes of students (Frisby et al., 2014). According to Weimer (2010), establishing a strong classroom rapport demands appreciating students’ ideas and perspectives. In a similar manner, Estepp and Roberts (2015) maintained that indicating concern for students’ welfare is vital for building a positive relationship in the class. In sum, a strong and positive emotional classroom rapport is characterized by teachers, who value students’ ideas and consider their well-being (Zhou, 2021).

### The outcomes of establishing a positive emotional classroom rapport

The establishment of a positive emotional classroom rapport between the teacher and students in the class has been scientifically approved to influence different academic domains such as enhanced classroom participation and engagement (Ibarra, 2014; Budzinska and Majchrzak, 2021; Xie and Derakhshan, 2021). It can also generate a positive interpersonal climate in the classroom and facilitate motivation, willingness to participate, and academic enjoyment (Weaver and Qi, 2005; Frisby et al., 2014). As pinpointed by Wilson and Ryan (2013), positive emotional classroom rapport is beneficial for both the teacher and students. It can improve students’ perceptions of learning, final grades, future job prospects, quality of classroom relationships, social interaction, and interpersonal communication skills (Wilson et al., 2010; Acharya, 2017). In addition to these benefits, positive emotional classroom rapport can create a safe space for students in which they do not apprehend to take part in academic activities and feel shamed in the classroom, which is explained in the following section.

### The definitions of student’s shame

Given its complexity and elusive nature, the concept of shame is not easy to define (Galmiche, 2018). Nevertheless, there is a consensus among educators that shame is a self-conscious feeling that entails reflection and evaluation of self (Salice and Montes Sánchez, 2016). This negative emotion is usually characterized by a sense of worthlessness, blushing, shrinking, sweating, increased heartbeat, stammering voice and so forth (Galmiche, 2018). As put by Tracy and Robins (2007), shame is an unpleasant emotion that is often accompanied by negative self-evaluation, enthusiasm to quit; and a sense of pain, distrust, helplessness, and insignificance. Lewis (1971), argued that shame is a broad negative feeling about the *self* as a consequence of some misdemeanor, shortcoming, or error. In simple terms, student’s shame is a sense of embarrassment in relation to what one has done/committed or what others perceive him/her.

### Typologies and cognates of shame

Like many other psycho-emotional constructs, student’s shame may come in different types and forms including transient (state) shame, chronic (trait) shame, internalized shame, toxic (debilitative) shame, and healthy (facilitative) shame (Benau, 2022). Drawing on the seminal works of Benau (2022) and Brandell and Ringel (2019) each type of shame is defined as what follows. According to these scholars, transient or state shame appears when an individual makes a minor mistake that usually passes quickly without causing problems in his/her life or career. On the other hand, chronic or trait shame is a more permanent and wounding form of shame that is omnipresent in all contexts and situations blocking one’s functionality and health.

Likewise, Cook (1996) defined internalized shame as a sense of humiliation and unworthiness that has permeated into one’s inner self making him/her internally shamed. Like internalized shame, toxic or debilitative shame is driven by one’s inner self (McFall and Johnson, 2009). However, this type of shame forms a part of one’s identity and it is a lasting state rather than a fleeting one. Individuals with toxic shame may try to display a perfect image of themselves in an attempt to hide how they feel inside (Mayer and Vanderheiden, 2019). Finally, healthy or facilitative shame may guide and serve the person toward self-correction and development (Mayer and Vanderheiden, 2019). Hence, it is more constructive than destructive for the person.

Regarding cognate terms, research shows that shame has been used synonymously or in place of some terminologies like guilt and embarrassment. Despite their seemingly similar faces, their meaning and scope differ. In simple words, shame highlights the inadequacy of the self, while guilt focuses on the badness of behavior. Moreover, shame encourages the disguise of deficiencies, whereas guilt inspires apology and rectifying the situation (Tangney et al., 2007). Another sibling concept is an embarrassment, which differs from shame in the sense that embarrassment is less intense and damaging, fleeting and temporary, and permeates into one’s public self rather than core identity or private self (Crozier, 2014).

### The consequences of EFL students’ shame

Students’ sense of shame in the classroom may bring about several problems for both students and teachers. In a seminal study, Galmiche (2018) enumerated some consequences of student’s shame including, high language learning anxiety, low cognitive functioning, low language proficiency, sense of worthlessness, loss of self-esteem, self-confidence, and self-concept, reduced psychological wellbeing, lowering working memory, unwillingness to communicate, and many more. In order to tackle these problems, EFL teachers and educational systems must try their best to establish a friendly academic environment for EFL students, first, to realize their shame and then through proper training and practices, enhance their self-regulatory strategies, self-esteem, and self-efficacy beliefs to become resilient and active agents of their learning.

### A control-value theory to classroom rapport and reduction of shame

CVT assumes that appraisals of control and values are pivotal to trigger the emergence of achievement emotions like shame (Pekrun, 2000). More specifically, CVT provides a framework for the analysis of the precedents and outcomes of learners’ emotions (Pekrun et al., 2005). Achievement emotions can be realized as either situational and temporal experiences or as trait incidents. Regarding the highlights of CVT, two types of appraisals are specified as the antecedents of achievement emotions: (a) subjective control of classroom tasks and (b) the perception of the values of these tasks. “Subjective control” indicates the perception of a learners’ sphere of influence over his or her activities (Pekrun, 2005). This means that from a CVT perspective, perceived control of classroom activities and holding a positive value of them are two determinants of positive achievement emotions (Pekrun and Perry, 2014). The literature on classroom rapport indicates that all the qualities created out of the positive and harmonious atmosphere of the classroom rapport can contribute to the controllability and value of the classroom activities. For instance, teachers’ emotional scaffolding (Zhang, 2021) embedded in the classroom rapport decreases negative emotions like shame and provides language learners with positive emotional experiences like enjoyment, which can consequently, broaden their attentional scope (Leung et al., 2019; Derakhshan et al., 2022b) and cognitive capacities (Fredrickson, 2001; Fredrickson, 2003). These benefits enable language learners to take control of their assigned tasks. As noted by Williams and Burden (1997), *shared intention* is a major aspect of classroom tasks in mediating their learning process. That is, whenever teachers’ and learners’ expectations are met, they feel competent and in control of the classroom tasks. The sense of mutual understanding, trust, and respect caused by the positive bond between teachers and student in classroom rapport build this shared intention. Besides, the immediacy and clarity that emerged *via* rapport in the class provide learners with a positive value for the classroom tasks (Derakhshan et al., 2022a).

Moreover, under the influence of the emotionally multimodal interaction between teachers and their students latent in classroom rapport, even some behaviors such as corrective feedback, which might be associated with negative emotions like anxiety and shame are positively acknowledged (see Bayat et al., 2020). On the other hand, due to the contagious nature of emotions (Hatfield et al., 1994), the transmitted emotions from teachers to students are reciprocated by their students (Shao and Parkinson, 2021). Thus, feeling their teachers’ emotional support, language learners attribute classroom tasks with positive values and see themselves as capable of coping with the challenges assigned for these tasks while being cognitively and affectively engaged in them. This engagement keeps them out of their resistance zone, where they experience shame and direct them towards a congruence zone where a relationship of trust and a positive atmosphere between teachers and learners are built. In these conditions, they are open to new experiences, regard classroom tasks as highly valuable, and feel in control of them. These high controllability and positive value determine their positive emotions of achievement (Shao et al., 2020). More specifically, the positive air of classroom rapport can provide language learners with a playful mindset, reflected in other-directed playfulness, *via* which they can shift from negative emotions like shame to positive emotions and openness to communicate (Barabadi et al., 2021).

### Final remarks

In this theoretical analysis, it was argued that EFL teachers’ ability to establish a positive emotional classroom rapport with their pupils can generate many positive academic outcomes such as self-disclosure (Hosek and Thompson, 2009), immediacy (Rocca, 2008), clarity (Chesebro and McCroskey, 1998), motivation (Zheng et al., 2021), success (Jimerson and Haddock, 2015), confirmation (Goodboy and Myers, 2008), sense of fulfillment (Nathan, 2018), instructional communication competence (Worley et al., 2007), and academic engagement (Zhu and Urhahne, 2021). Besides these positive constructs, classroom rapport can hinder the emergence and development of negative emotions in students, too. One such emotion among EFL students is shame, which have received scant attention from L2 scholars to date. The shortcoming in researching this construct is that the current body of research has been limited to unraveling the sources and consequences of student’s shame in L2 classes. Furthermore, the role of positive constructs like classroom rapport in minimizing or turning this negative emotion into positive feelings like resilience and engagement has been both theoretically and empirically overlooked thus far. To break the ice, the present study made an effort to theoretically connect two constructs of positive emotional classroom rapport and shame. While these variables have been studied separately, their interplay and the preventive role of positive emotional classroom rapport in curbing the other construct have been left uncharted. This study contends that through proper strategies and techniques, EFL teachers can build a positive emotional classroom rapport with their students, boost their academic performance, and simultaneously, minimize the emergence and growth of negative emotions and setbacks. More specifically, to prevent student’s shame, EFL teachers can bring to light the criticality of interpersonal interactions in the class *via* changing the classroom culture/climate into a positive one, confirming students’ performances and behaviors, attending to students’ questions, praising students, regulating teaching style to students’ needs, wants, and goals, using humor in the class, using self-disclosure, promoting interpersonal communication skills such as clarity, credibility, immediacy, and stroke, and finally, integrating “loving pedagogy” into EFL education, which facilitates the establishment of positive emotional classroom rapport as the blocker of student’s shame.

In light of these promising insights, this theoretical review can have implications for EFL teachers, teacher educators, school principals, and researchers. EFL teachers may find this review article helpful in that it can enrich their knowledge and awareness of the interpersonal aspect of EFL education and the linkage of teachers’ and students’ emotions in the classroom. They can also use the suggested strategies in their own classes to prevent other student’s negative emotions (e.g., boredom, shyness, hopelessness, and disengagement). Teacher trainers can also take advantage of ideas put in this article in the sense that they can design and offer workshops and training courses to EFL teachers focusing on different self-regulatory strategies that L2 teachers can use to prevent students’ psycho-emotional problems. Likewise, school principals may benefit from this study in that they can realize the significance of school and classroom culture and climate in generating and preventing different outcomes in academia. The final group for which this review may be advantageous is L2 researchers in that they can detect the existing gaps in this domain and run complementary studies to make the picture clearer. More precisely, this line of research has only focused on the causes and consequences of student’s shame, hence future studies can be done on solutions for these problems. As a case in point, empirical studies can be conducted on the integration of positive psychology or loving pedagogy in preventing or reducing EFL students’ shame in the classroom. Another gap identified in this study concerns the role of cultural backgrounds in the formation and function of the three variables reviewed. Therefore, it is an interesting idea to explore these constructs in different cultural contexts to see if students and teachers from other contexts perceive and tackle them in a similar or different way.

Future research is recommended using different theoretical perspectives in exploring EFL teachers’ positive emotional classroom rapport and students’ classroom shame. An outstanding theory is Dynamic Systems Theory (DST), which argues that the behavior of organic systems is not the outcome of causal relationships between static elements, but instead an ongoing interaction among all agents in the system (i.e., EFL students and teachers) (Larsen-Freeman, 1997). DST fits with the persistent flux common in classroom interactions and students’ socio-emotional factors (e.g., silence and shame). Finally, enthusiastic researchers are suggested to use qualitative and longitudinal research designs like ethnography, phenomenology, diary, and journals to track the dynamism of classroom rapport, silence, and shame.

## Data availability statement

The original contributions presented in the study are included in the article/supplementary material, further inquiries can be directed to the corresponding author.

## Author contributions

CK took responsibility of research design and wrote the manuscript. JW proofread the manuscript. All authors contributed to the article and approved the submitted version.

## Funding

Acknowledgment Research Team on Vocational Education Development Tracking in United States, Canada and the World Bank; Teaching Reform and Practice of Higher Vocational Colleges English Curriculum Ideological and Political Education in the New Era.

## Conflict of interest

The authors declare that the research was conducted in the absence of any commercial or financial relationships that could be construed as a potential conflict of interest.

## Publisher’s note

All claims expressed in this article are solely those of the authors and do not necessarily represent those of their affiliated organizations, or those of the publisher, the editors and the reviewers. Any product that may be evaluated in this article, or claim that may be made by its manufacturer, is not guaranteed or endorsed by the publisher.
